# Supplementary material for: A direct RT-qPCR approach to test large numbers of individuals for SARS-CoV-2
Source: PLoS One. 2020 Dec 31;15(12):e0244824. doi: 10.1371/journal.pone.0244824 (PMC7774962; doi:10.1371/journal.pone.0244824)
Supplement: S1 Table — (DOCX) [file pone.0244824.s001.docx]

|  | | **Number of positive reactions/Number of tests** | |
| --- | --- | --- | --- |
| **RNA copies per 2 µl RT-qPCR reaction** | **RNA copies/mL (mouthwash)** | **first RT-qPCR** | **second RT-qPCR** |
| 100 | 50,000 | 3/3 | ND |
| 20 | 10,000 | 3/3 | ND |
| 14 | 7,000 | ND | 20/20 |
| 10 | 5,000 | 3/3 | 20/20 |
| 8 | 4,000 | ND | 20/20 |
| 6 | 3,000 | ND | 19/20 |
| 2 | 1,000 | 0/3 | ND |
| 0.4 | 200 | 0/3 | ND |
| 0.08 | 40 | 0/3 | ND |

**Supplementary Table 1. Analytical limit of detection of the gurgle NEB SARS-CoV-2 assay.** Experimental limit of detection was determined as the lowest concentration tested with 95% detection. Each 25 µl reaction contained 2 µl of a pooled mouthwash and differing amounts of a non-replicative recombinant virus from the AccuPlex SARS-CoV-2 Verification Panel (0505-0129).
