# Supplementary figures and images for: A direct RT-qPCR approach to test large numbers of individuals for SARS-CoV-2
Source: PLoS One. 2020 Dec 31;15(12):e0244824. doi: 10.1371/journal.pone.0244824 (PMC7774962; doi:10.1371/journal.pone.0244824)

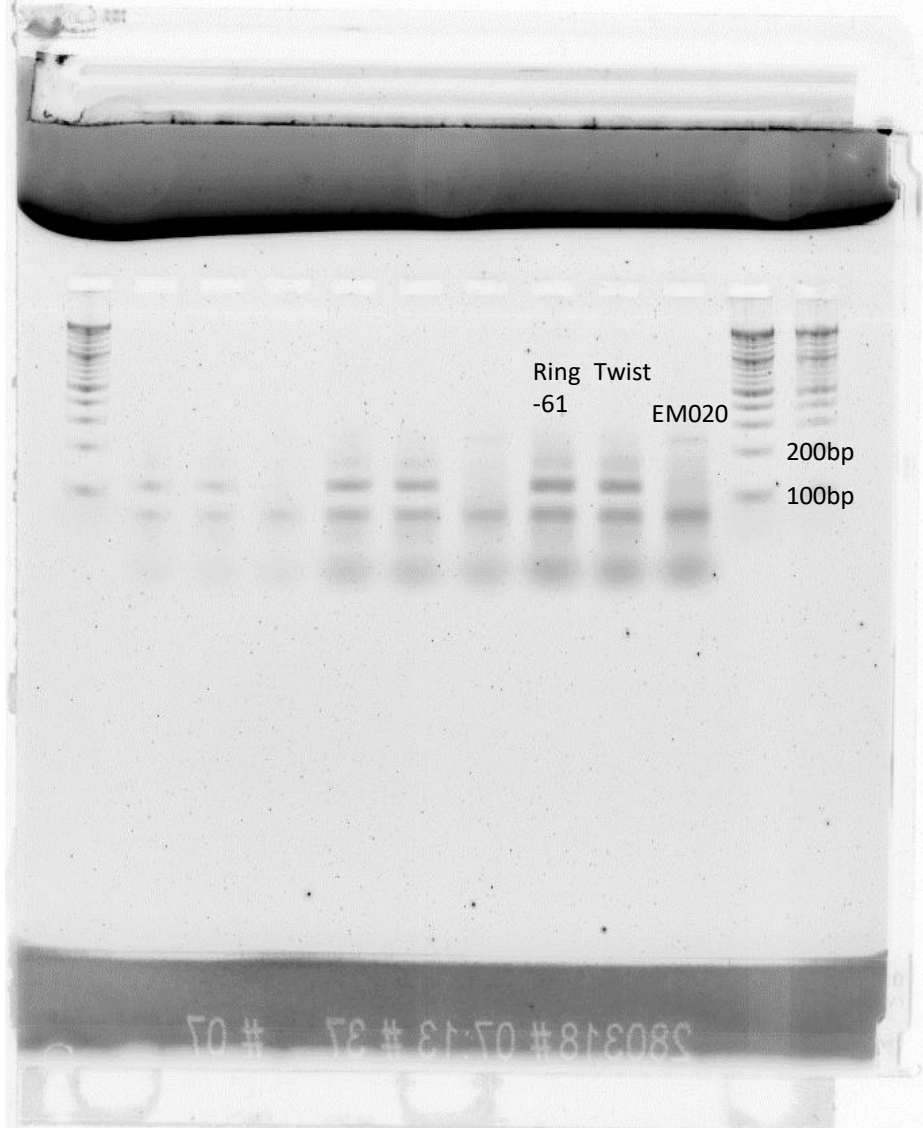

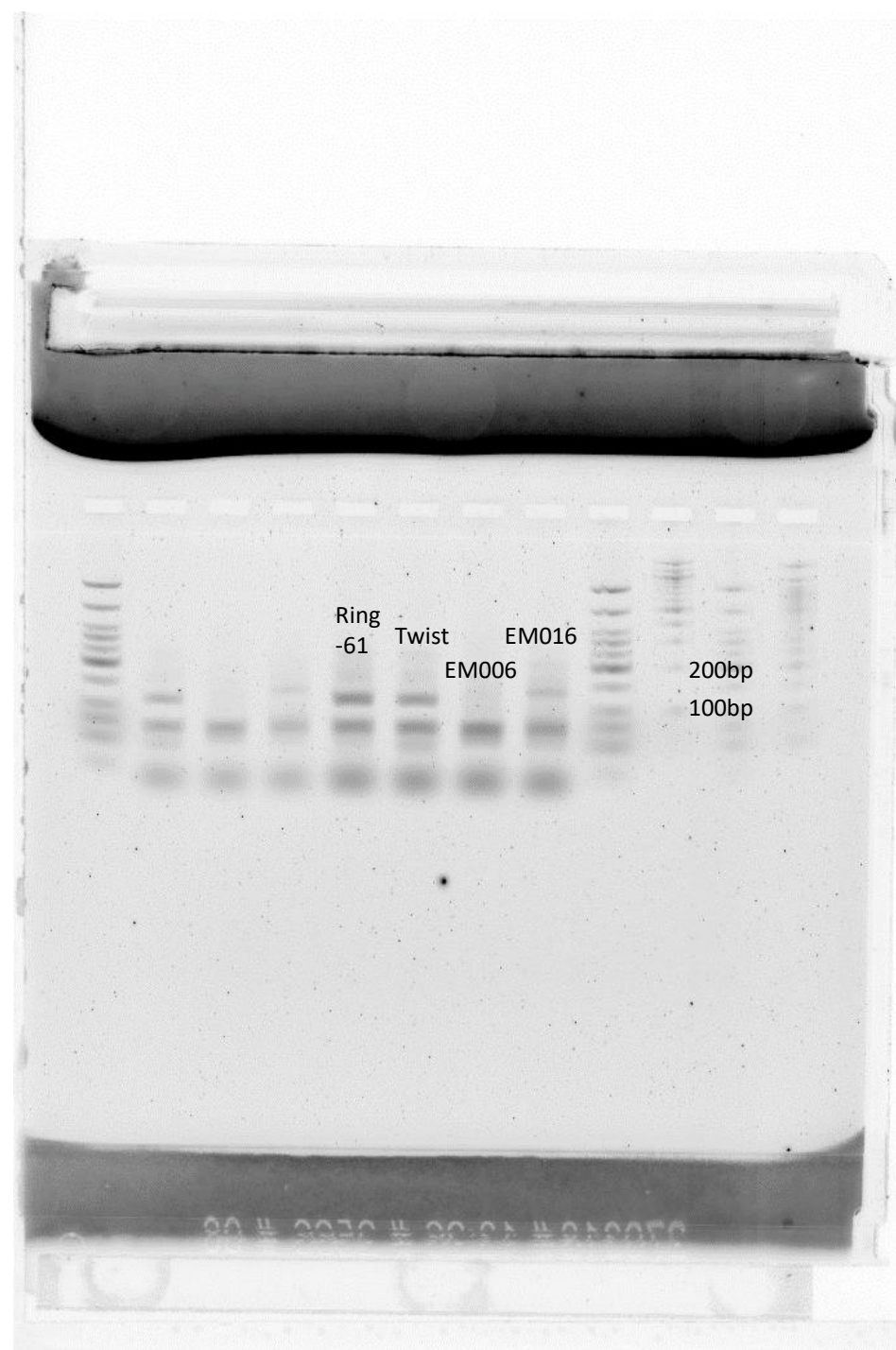

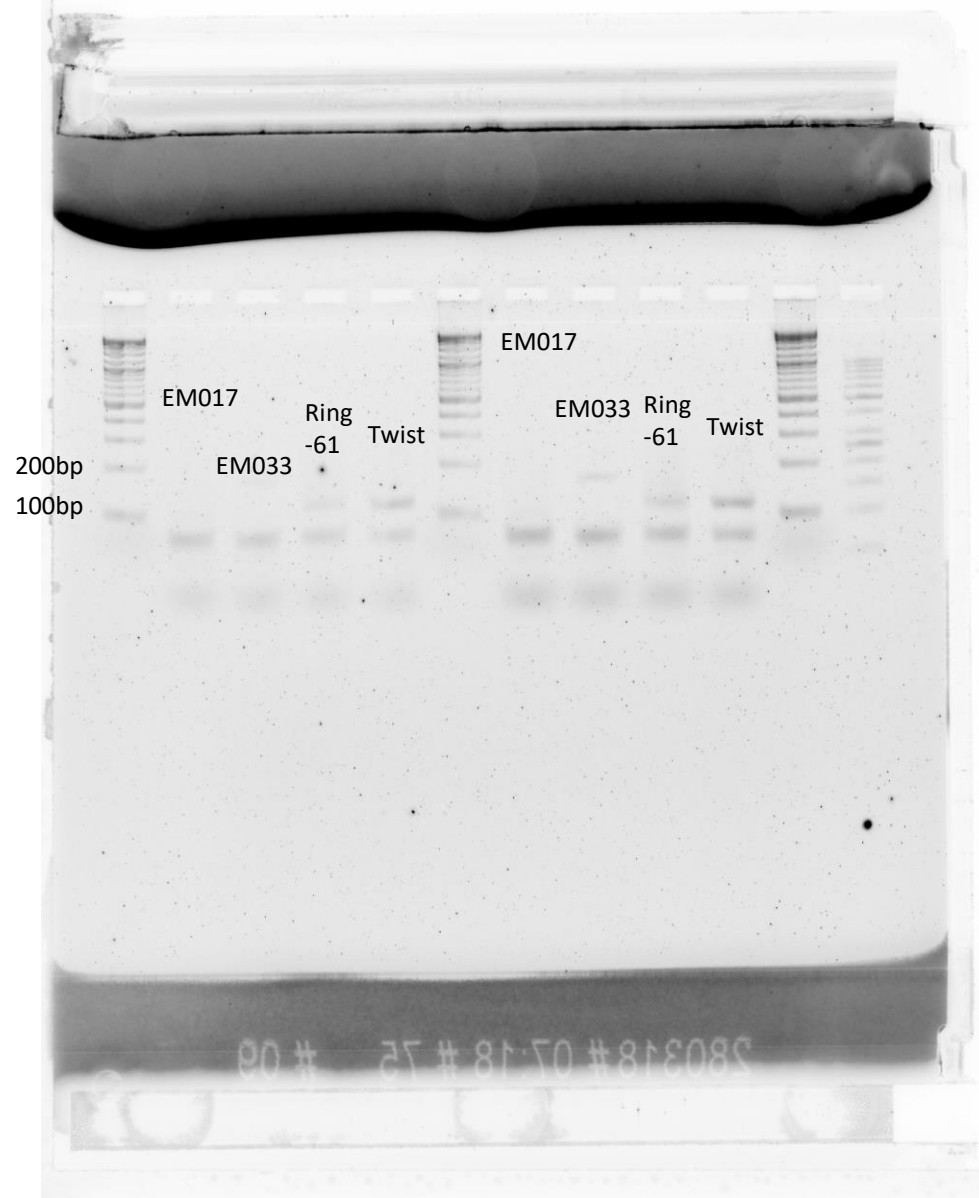

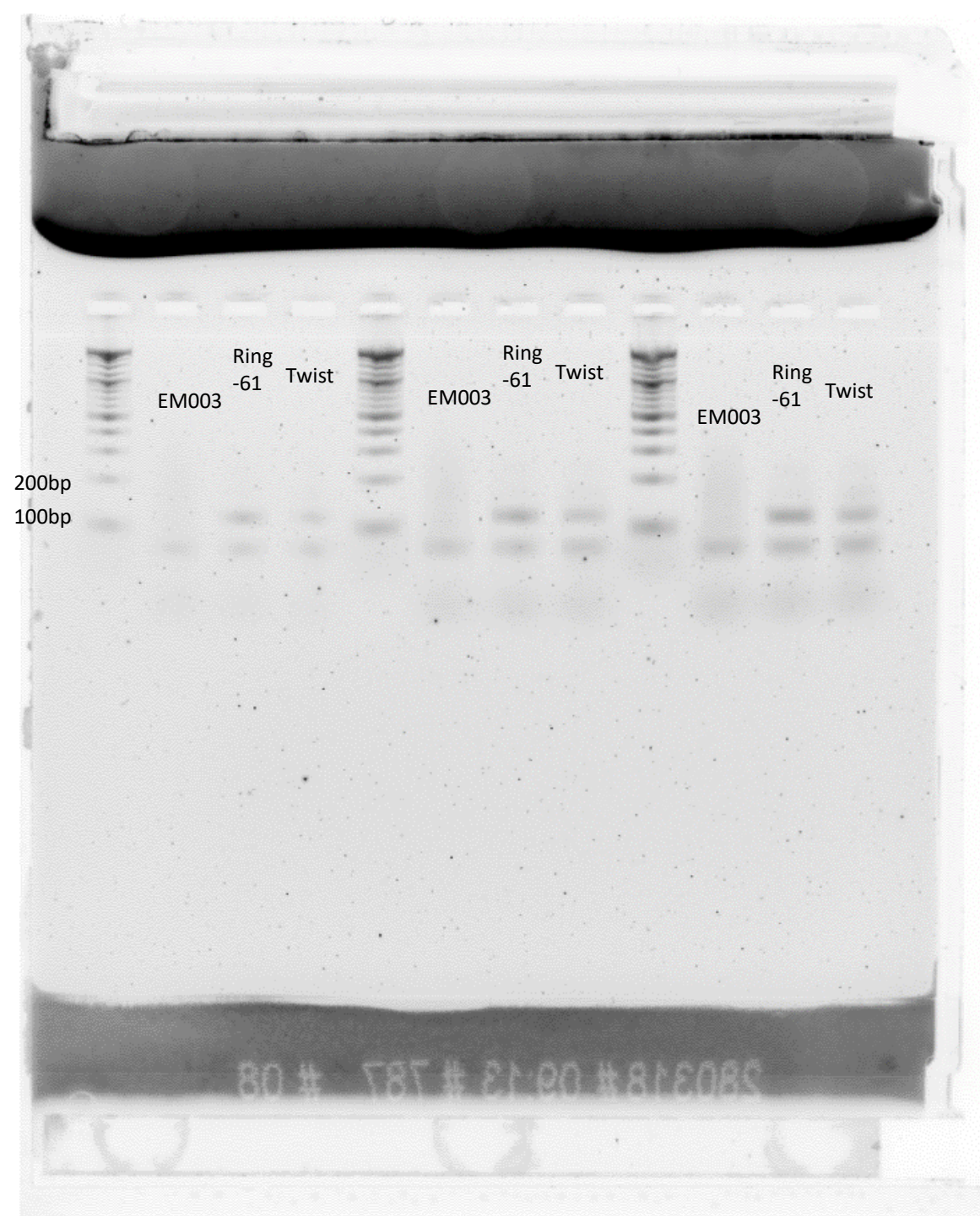

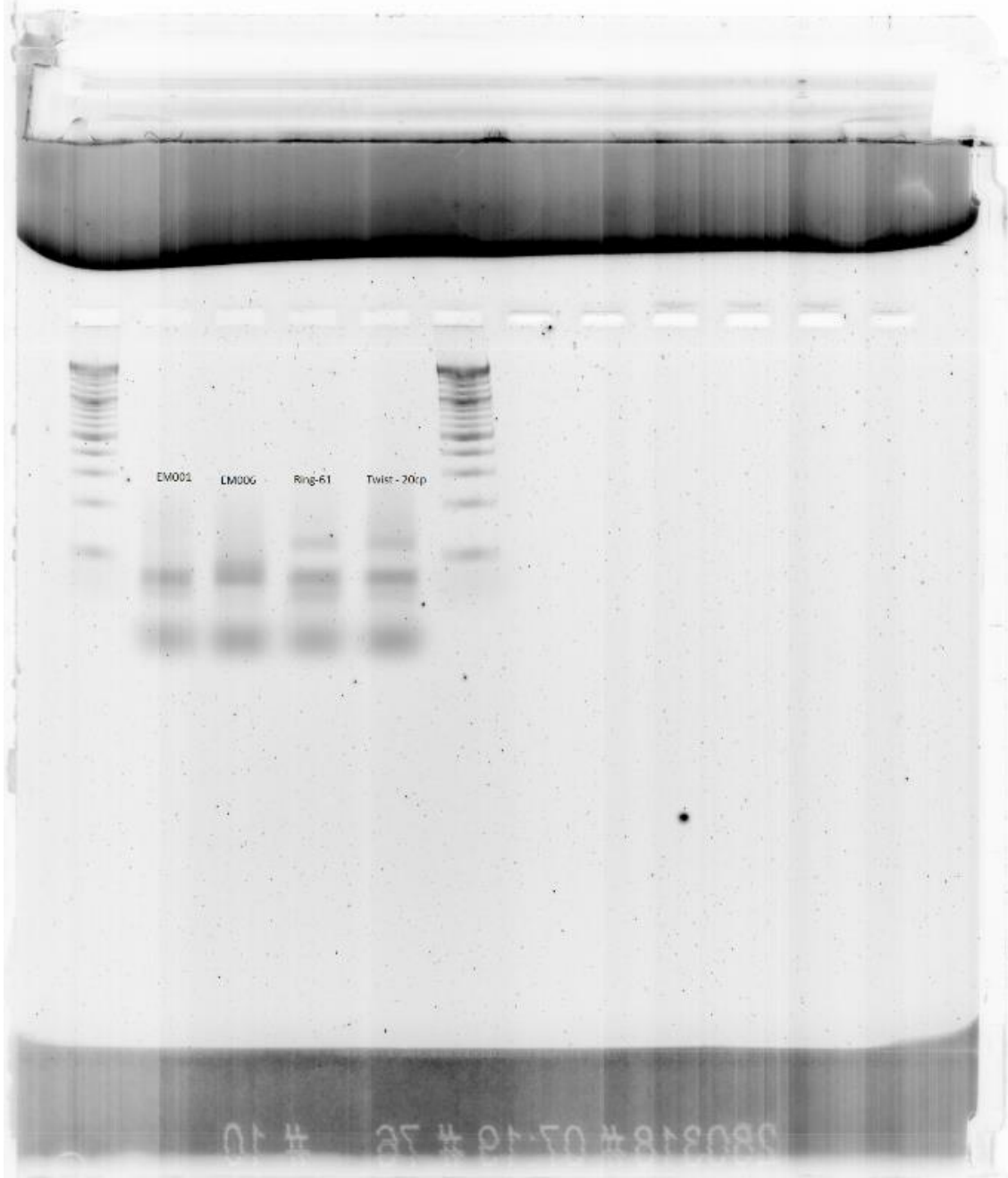

01 # 25 # 01-50 # 818080

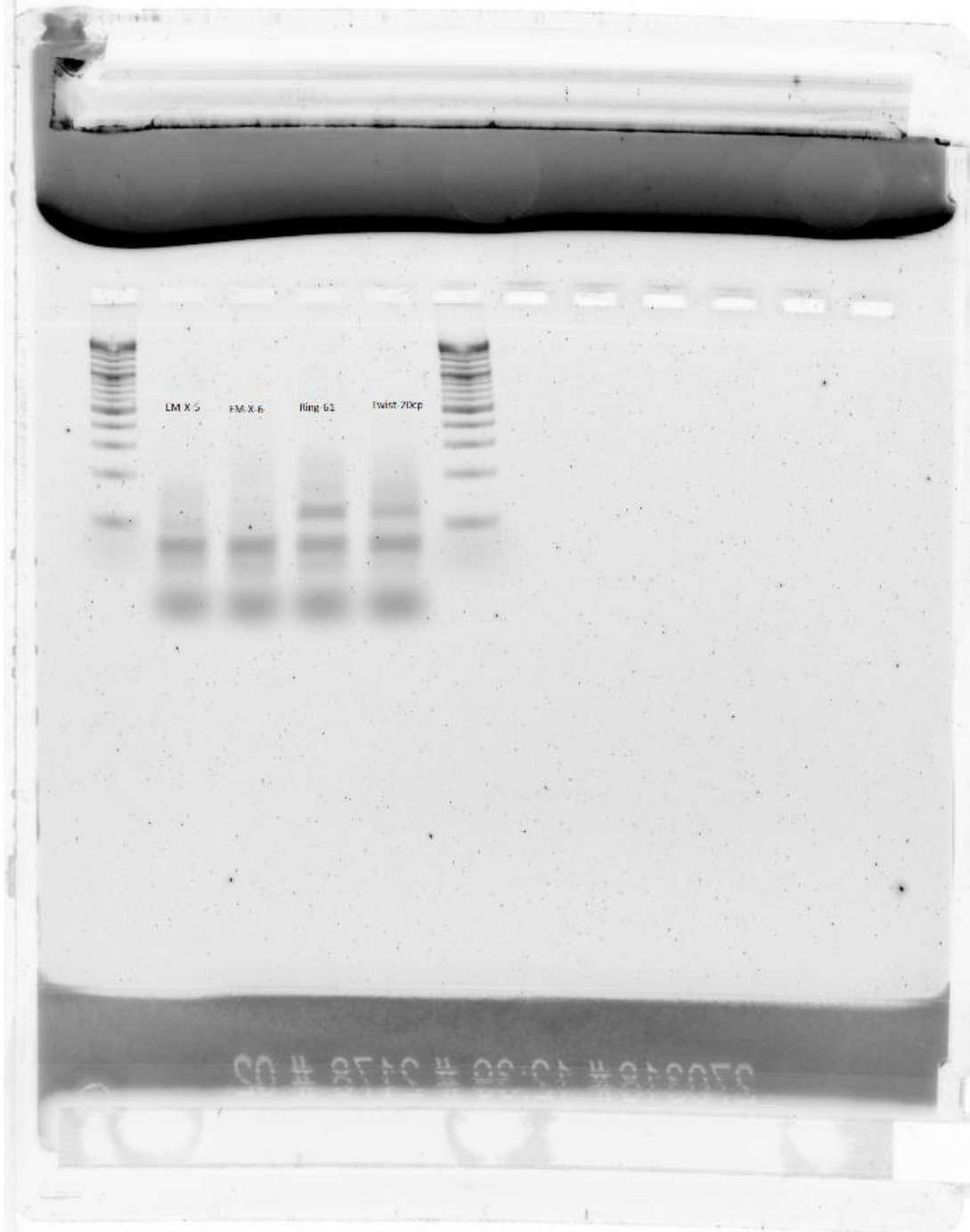

Supplement: S1 Raw images — (PDF) [file pone.0244824.s003.pdf]
