# Supplementary material for: A direct RT-qPCR approach to test large numbers of individuals for SARS-CoV-2
Source: PLoS One. 2020 Dec 31;15(12):e0244824. doi: 10.1371/journal.pone.0244824 (PMC7774962; doi:10.1371/journal.pone.0244824)
Supplement: Attachment — Submitted filename: Response to Reviewers.docx [file pone.0244824.s004.docx]

6. Review Comments to the Author

Please use the space provided to explain your answers to the questions above. You may also include additional comments for the author, including concerns about dual publication, research ethics, or publication ethics. (Please upload your review as an attachment if it exceeds 20,000 characters)

Reviewer #1: The authors have addressed my comments of review round #1. The comments regarding the validation of the RT-PCR protocol raised by reviewer #2 have been addressed in the response to reviewer letter by the authors.

Reviewer #2: I would like to thank the authors for revising their manuscript. The authors have partly addressed my comments, and I still have 2 remaining comments:

1. In their responses to the other reviewers and my comments, the authors state that their proposed protocol is for SARS-CoV-2 surveillance. However, I think the authors have mixed up the meaning of surveillance and screening, which refer to different types of tests in epidemiology (https://www.fda.gov/medical-devices/coronavirus-covid-19-and-medical-devices/covid-19-test-uses-faqs-testing-sars-cov-2). Surveillance is applied to the population level and not to report results back to the individual level, while screening refers to testing of individuals without symptoms. Thus, the authors should use the proper terminology throughout their manuscript.

We thank the reviewer for clarifying for us. We now talk exclusively about screening (and not surveillance) in the manuscript.

2. In my previous review I commented on the lack of an extensive sensitivity analysis for the current protocol. I agree with the authors that screening assays may need a different level of sensitivity as compared to diagnostic assays. However, sensitivity analysis is important to enable comparisons between assays to help labs make a decision for an assay. For rapid tests and at-home test I think lower sensitivity is justified, but considering that the current assay is PCR-based and that other extraction-free (and saliva-based) PCR assays are available with a seemingly higher sensitivity I wonder what the added value of the current assay is for other labs. I urge the authors to 1) determine the limit of detection of their assay (see example guidelines for LOD here: https://www.fda.gov/media/135659/download) and 2) to add a “limitations of the study” section to their discussion to provide a rational for other labs to use this assay (as compared to other more sensitive extraction-free and non-swab based PCR assays).

1) We have now performed the experiment suggested by the reviewer and we write the following in Results: “Next we tested the smallest number of SARS-CoV-2 RNA molecules that can be detected in the assay. We find that six RNA molecules can be detected in 19 out of 20 replicate reactions, which corresponds to limit of detection of 3,000 RNA molecules per milliliter of mouthwash (Suppl. Table 2).”

Since we were able to detect only six RNA molecules the assay is very close to the theoretical limit of detection of single viral RNA molecules. The factor that limits the experimental limit of detection is the volume of the mouthwash that we can use in the assay without inhibiting the reaction. This limits also other extraction-free assays. We are not aware of any other direct assays that are substantially more sensitive.

2) We have added a last section called “Limitations and Practical Considerations” after the Discussion section (line 196 ff). It discusses the lower sensitivity and the usefulness of the assay and draws attention to these issues more than when this discussion was embedded in the Discussion.

Reviewer #3: What is the purpose of Figure 4? It's unclear, lacks a time line, and is adequately handled in the text. Consider omitting.

We agree that the figure is simplistic. Yet, we find it useful for illustrating a practical scheme as it implemented currently in our institute and at the retirement home. If possible, we would like to keep in the manuscript.
